# Duration Versus Magnitude of BIS-Measured EEG Suppression and Postoperative Recovery Patterns: A Prospective Observational Study

**DOI:** 10.3390/jcm15030975

**Published:** 2026-01-26

**Authors:** Ahmet Yuksek, Bedirhan Gunel, Ayşe Zeynep Turan Civraz

**Affiliations:** Department of Anesthesiology and Reanimation, Kocaeli City Hospital, Kocaeli 41060, Turkey; bedirhangunel71@gmail.com (B.G.); ayse.zeynep@gmail.com (A.Z.T.C.)

**Keywords:** anesthesia, bispectral index suppression, emergence agitation, neuromonitoring, processed EEG

## Abstract

**Background**: This study aimed to determine whether the duration or the magnitude of intraoperative BIS suppression has a greater impact on postoperative recovery. **Methods**: In this observational study, 141 patients were monitored for BIS values, suppression ratio (SR), maximum suppression ratio (SRmax), and total suppression time (SRT) during the perioperative period. Recovery phenotypes were assessed using the Richmond Agitation-Sedation Scale (RASS). Statistical analyses evaluated the relationship between BIS suppression parameters (SR, SRmax, SRT) and postoperative sedation or emergence agitation. Optimal thresholds for clinically significant suppression were determined. **Results**: Patients classified into the sedation group according to RASS scores exhibited significantly higher intraoperative SRmax values (*p*: 0.038) and prolonged SRT (*p*: 0.001) compared to the agitated group. An SRT ≥ 7.5 min predicted sedated recovery with 86.7% sensitivity and 39.4% specificity (AUC = 0.651, 95% CI: 0.561–0.742, *p*: 0.002). Similarly, an SRmax ≥ 19.5 was associated with sedated recovery (85.3% sensitivity, 53.0% specificity; AUC = 0.683, 95% CI: 0.592–0.775, *p*: 0.001). No significant association was found between BIS suppression and emergence agitation. **Conclusions**: Prolonged intraoperative BIS suppression and higher SRmax values are comparably predictive of postoperative sedation but not agitation. Monitoring these parameters may aid in anticipating recovery patterns.

## 1. Introduction

Bispectral Index (BIS) is a neurophysiological measurement that enables the monitoring and quantification of the depth of anesthesia or sedation in patients undergoing surgery or certain medical procedures. It helps healthcare professionals assess the level of consciousness and adjust anesthesia or sedation accordingly to ensure patient safety and optimal outcomes [1,2]. In addition to its role in monitoring anesthesia depth, the bispectral index has also been found to be useful in detecting anesthesia-related cerebral hypoperfusion, optimizing sedation, and helping to shorten the duration of weaning, ICU stay, and overall healthcare costs [3].

BIS has played a crucial role in the emergence of advanced monitoring technologies in anesthesia [4]. Its ability to provide real-time assessment of consciousness and anesthesia depth has greatly improved patient care and safety in surgical and medical settings. Similar to other processed electroencephalographic monitors, BIS has been shown to decrease the risk of awareness when integrated into anesthetic titration strategies [5]. The ideal perioperative BIS range is generally considered to be between 40 and 60, indicating an adequate level of anesthesia or sedation. Lower BIS values are associated with deeper levels of anesthesia, while higher BIS values suggest lighter sedation [6]. Although it is best to avoid unnecessary suppression of the brain, in some cases, hypothermia-induced slowing of electrical brain activity, reduced cerebral perfusion due to hypotension, or burst suppression related to deep anesthesia may occur as perioperative adverse events. These changes can be monitored using raw EEG recordings as well as the processed EEG provided by the Bispectral Index [7].

BIS Suppression Ratio (BIS-SR) is a parameter derived from BIS that measures the degree of burst suppression in the electroencephalogram. Although it is generally known that excessive suppression is associated with adverse postoperative outcomes, its relationship with recovery patterns has not been sufficiently investigated [8]. Similar to perioperative hypothermia, BIS suppression is a parameter that should be avoided; however, it can occur unintentionally [9]. The precise threshold at which the associated risk increases significantly has not yet been clearly defined.

This study investigates the relationship between intraoperative BIS-SR and emergence agitation (EA) or sedated emergence (ES) in patients undergoing lumbar spine surgery. In our study, we test the hypothesis that monitoring the maximum BIS suppression value and the total BIS suppression time can predict the phenotype of recovery after anesthesia.

## 2. Materials and Methods

### 2.1. Study Design and Setting

For our prospective observational study, ethics committee approval numbered KAEK-2023-32 was received from Kocaeli City Hospital Clinical Research Ethics Committee on 23 December 2023, in accordance with the Declaration of Helsinki. Additionally, an international study registration was performed on 21 March 2024, prior to participant enrollment (Clinicaltrials.org; NCT06334939). Patient recruitment and data collection were conducted between 1 April and 1 August 2024. Written consent for participation and publication was obtained from all patients participating in the study. The study patients were informed before the operation and patients with informed consent were included in the study. We structured our article in accordance with CONSORT guidelines.

### 2.2. Participants

**Figure 1:**
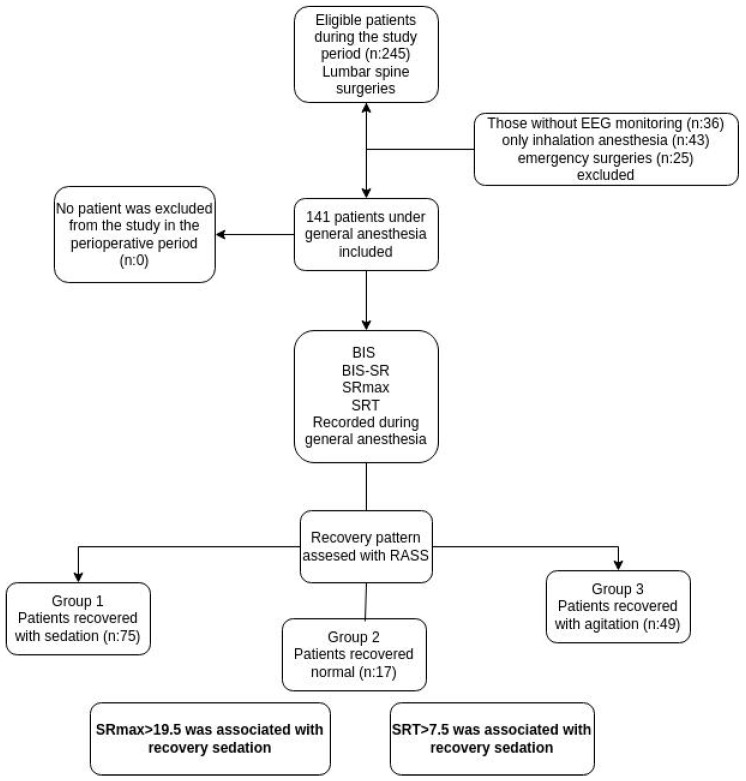
Flow chart.

The study included patients who underwent lumbar spine surgery under general anesthesia in the operating rooms of Kocaeli City Hospital, a tertiary hospital, between January 2024 and July 2024. Only patients aged 18 years and older were included. Among them, those who received Total Intravenous Anesthesia (TIVA) as the anesthetic technique and those who were monitored using the BIS monitor (GE BIS Module, GE Healthcare, Chicago, IL, USA) were included in the study. Patients whose anesthesia maintenance was completed solely with inhalational anesthesia were excluded from the study for standardization purposes. It was planned to exclude hemodynamically unstable patients and patients requiring vasoactive drugs from the study. The workflow is illustrated in the flowchart (Figure 1).

Variables: the variables recorded in the study were anesthesia duration, BIS, BIS-SR, maximum suppression value (SRmax), total suppression time (SRT), postoperative eye opening time, EA or ES according to the Richmond agitation sedation scale (RASS).

Anesthesia duration was defined as the total time between the induction of anesthesia and the extubation of the patient. In patients monitored preoperatively, hemodynamic data and data obtained with the BIS monitor were recorded at 10-min intervals during the operation. BIS values are parameters formed by the analysis of EEG waves and presented as numerical intervals. The suppression values ware calculated as follows:

Real time SRmax and SRT values were calculated using the method in the Balanced anesthesia study which involving 6644 patients [8]. In this method; SRmax represents the highest suppression value that occurs during the operation. The SRT consists of the sum of the minutes when the suppression ratio value is greater than zero. The timer was started when the BIS-SR value was greater than zero and stopped when the BIS-SR was zero. This period was repeated as the total suppression duration for each BIS-SR> 0 episode. In addition, the SRT and SRmax were recorded. The time from the termination of TIVA to the patient’s eye-opening was recorded as the eye opening time.

EA was assessed using the RASS. According to the scale, patients were assessed with scores ranging from −5 to +4, with 0 points being calm, +1 being restless, and +2 points and above being agitated. In the postoperative recovery unit, patients with RASS <−1 were included in the analysis as sedated (Group 1) and patients with >+2 were included in the analysis as agitated (Group 3). Patients with a RASS score of 0, indicating a calm state, were classified as Group 2.

### 2.3. Data Sources/Measurements

Propofol and remifentanil were preferred for TIVA, which was manually adjusted. Target BIS values between 40 and 60 were aimed to be kept after standard anesthesia induction. Following induction, neuromuscular blocking agents were intentionally avoided during maintenance anesthesia to preserve the reliability of intraoperative neuromonitoring, including motor-evoked and somatosensory-evoked potentials. Adequate immobility and anesthetic depth were ensured through close titration of hypnotic and opioid agents, guided by hemodynamic parameters, neuromonitoring feedback, and BIS values. The lowest and highest values outside the target range for intraoperative BIS values were recorded perioperatively. SRT and SRmax were also documented on the study form. Routine BIS values at 10-min intervals and real time SR values were also recorded during the perioperative period.

There were no hemodynamically unstable patients intraoperatively, nor were there any patients who required inotropic drugs. It was planned to exclude these patients from the study.

At the end of the operation, patients received 100 mg of tramadol for postoperative analgesia as standard. TIVA was terminated at the end of the surgery. The time taken for extubation after the end of anesthesia, the time between the extubated patients’ arrival to the recovery room, and the agitation/sedation times in the recovery room were recorded. In addition, eye-opening times were compared between the groups. Recovery agitation scores were made by the 2nd anesthesiologist in the recovery unit who was unaware of the patients’ perioperative follow-ups. In this way, the patients’ recovery processes were evaluated independently.

### 2.4. Study Size

A priori sample size calculation was based on data from a pilot study conducted with 60 patients. The incidence of emergence agitation (EA), defined as a RASS score > +1 at the 10th minute following extubation, was used as the primary outcome. Based on the effect size observed in the pilot data, it was estimated that a minimum of 90 patients would be required to achieve a power of 95% with a two-tailed alpha level of 0.05. The sample size calculation was performed using G*Power software (version 3.1), and the estimation was also supported by findings from a previous study [10] comparing EA rates between inhalational and TIVA anesthesia groups.

### 2.5. Statistical Analysis

Statistical analyses were performed using IBM SPSS Statistics version 22.0 (IBM Corp., Armonk, NY, USA). Continuous variables were expressed as median and interquartile range (IQR), while categorical variables were presented as number (n) and percentage (%). The distribution of continuous variables was assessed using the Shapiro–Wilk test, which indicated non-normal distributions.

Comparisons among the three recovery groups (sedated emergence, normal recovery, and emergence agitation) were conducted using the Kruskal–Wallis test for continuous variables. When a statistically significant overall difference was identified, pairwise post hoc comparisons were performed using the Mann–Whitney U test with appropriate adjustment for multiple comparisons, and adjusted *p*-values were reported. Comparisons of categorical variables, including sex distribution, were performed using the chi-square test.

Receiver operating characteristic (ROC) curve analyses were conducted to evaluate the ability of maximum suppression ratio (SRmax) and suppression ratio duration (SRT) to discriminate recovery profiles. The area under the curve (AUC) with 95% confidence intervals, sensitivity, specificity, and the Youden index were calculated to determine optimal cutoff values. A two-sided *p*-value < 0.05 was considered statistically significant.

## 3. Results

### 3.1. Patient Characteristics

During the study period, 245 patients underwent spinal surgery. Of these, patients without EEG monitoring (*n* = 36), those maintained with inhalational anesthesia (*n* = 43), and those who underwent emergency procedures (n = 25) were excluded. None of the eligible patients declined to participate, and no intraoperative exclusions due to hemodynamic instability occurred. A total of 141 patients were included in the analysis. According to recovery characteristics, 75 patients (53.2%) were classified as recovery with sedation (Group 1), 17 patients (12.1%) as normal recovery (Group 2), and 49 patients (34.8%) as emergence agitation (Group 3).

The median age of the overall cohort was 50 years (IQR 21), with no statistically significant difference among the three groups (*p* = 0.235). Sex distribution differed significantly between groups (*p* = 0.007), with a higher proportion of female patients in Group 1, whereas Group 3 included a higher proportion of male patients (Table 1).

Preoperative BIS values were comparable across groups, with median values of 88 (IQR 14), 87 (IQR 5), and 88 (IQR 10.75) in Groups 1, 2, and 3, respectively (*p* = 0.903). Similarly, no significant differences were observed in intraoperative minimum BIS values (median 36–38) or maximum BIS values (median 57–59) among the groups (*p* = 0.154 and *p* = 0.364, respectively).

In contrast, suppression-related variables differed significantly between recovery groups. The median maximum suppression ratio (SRmax) was significantly higher in Group 1 [28 (IQR 12)] compared with Groups 2 [18 (IQR 20)] and 3 [18 (IQR 18.5)] (*p* < 0.001). Total suppression time (SRT) also differed among groups (*p* = 0.004), with higher median values observed in Group 1 [12 (IQR 8)] compared with Group 3 [10 (IQR 9)], while Group 2 showed similar values [12 (IQR 11)] (Table 1). Operation duration varied significantly between groups (*p* = 0.011). The longest median operation time was observed in Group 1 [168 min (IQR 50)], whereas shorter durations were recorded in Group 2 [150 min (IQR 62.5)] and Group 3 [145 min (IQR 50)].

Median propofol dosage did not differ significantly among the groups (*p* = 0.100), with values of 108 mg (IQR 22), 98 mg (IQR 27), and 103 mg (IQR 25.5) in Groups 1, 2, and 3, respectively. Likewise, remifentanil infusion rates were comparable across groups (*p* = 0.092).

Recovery time differed significantly among the three groups (*p* = 0.001). Group 1 exhibited a longer median recovery time [13 min (IQR 6)] compared with Group 2 [10 min (IQR 6.5)] and Group 3 [10 min (IQR 9)] (Table 1). Post hoc pairwise comparisons using the Mann–Whitney U test with adjustment for multiple comparisons revealed that SRmax and recovery time were significantly higher in Group 1 compared with Group 2 (adjusted *p* = 0.007) and Group 3 (adjusted *p* = 0.015). In contrast, no significant differences were observed between Group 2 and Group 3 (adjusted *p* = 0.849).

### 3.2. ROC Analysis for Emergence Agitation and Sedation

According to the ROC analysis, both SRmax and SRT demonstrated statistically significant discriminative ability in predicting RASS score categories. For the SRmax variable, the area under the curve (AUC) was 0.683 (95% CI: 0.592–0.775, *p* < 0.001). Similarly, SR duration yielded an AUC of 0.651 (95% CI: 0.561–0.742, *p* = 0.002).

**Figure 2:**
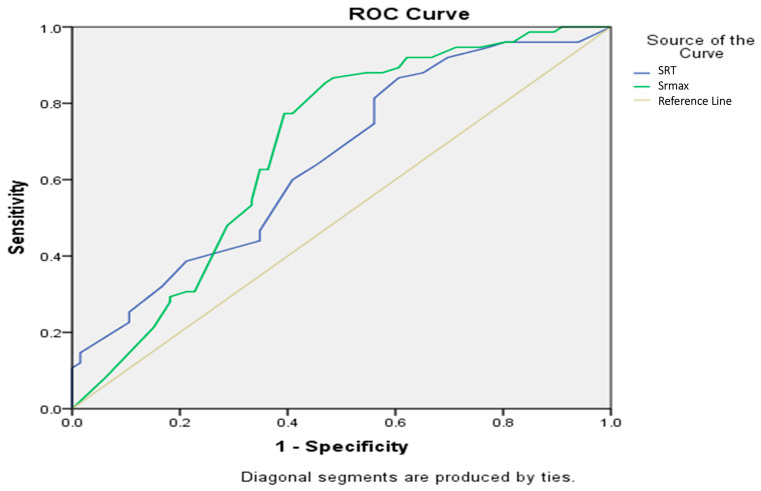
ROC analysis for the predictive power of SRmax and SRT on recovery with sedation, Srmax; maximum suppression value, SRT; total suppression time. SRmax value ≥ 19.5 provided the highest Youden Index (J = 0.383), with a sensitivity of 85.3% and a specificity of 53.0%. SR duration ≥ 7.5 was identified as optimal, yielding a sensitivity of 86.7% and a specificity of 39.4% (J = 0.261).

Based on cut-off analysis, an SRmax value ≥ 19.5 provided the highest Youden Index (J = 0.383), with a sensitivity of 85.3% and a specificity of 53.0%. For SR duration, a cut-off of ≥7.5 was identified as optimal, yielding a sensitivity of 86.7% and a specificity of 39.4% (J = 0.261). These findings suggest that both SRmax and SRT may contribute meaningfully to predicting emergence profiles based on RASS scores (Figure 2).

## 4. Discussion

This study examined the effects of perioperative EEG suppression measured by the BIS Suppression Ratio (BIS-SR) on emergence agitation and sedation. Patients with a higher intraoperative EEG suppression burden, reflected by a higher BIS-SR and longer suppression duration, exhibited higher sedation scores during the recovery period. An EEG suppression duration exceeding 17 min or a BIS-SR greater than 26.5 was associated with a sedated recovery. Both SR duration and SRmax showed comparable predictive performance for RASS-defined sedation during recovery. Neither the duration nor the magnitude of EEG suppression was associated with emergence agitation.

EA is characterized by unintended movements and limited cooperation, typically manifesting during the early stages of recovery from anesthesia. Consequently, patients may be at risk of developing undesirable complications, including self-harm, self-extubation, catheter dislodgement, involuntary increases in blood pressure, and heightened oxygen consumption. Emergence agitation is influenced by a variety of factors, including patient-specific characteristics such as age and sex, as well as the duration and type of surgery [11]. Additionally, anesthetic techniques, preoperative conditions, and postoperative pain management strategies may also contribute to the occurrence and severity of this phenomenon. Its incidence is given in a wide range in studies, and Lee et al. [12] also reported rates as high as 90.5%. Although it has short-term effects that are often neglected or ignored in patients, it is a reality that can also have serious consequences.

Early recognition and intervention are crucial due to the potential negative outcomes associated with excessive agitation or mobility, which may result in harm to the patient or medical staff, bleeding at the surgical site, accidental removal of drains or catheters, wound dehiscence, falls from the bed, displacement of vascular access, non-compliance with treatment, and an overall increase in treatment costs [13,14]. Risk factors for emergence agitation (EA) include male sex, type of surgery, emergency procedures, the anesthetic agents used, patient pain levels, and the presence of a urinary catheter. While some of these factors can be modified, others are beyond control. Nevertheless, the occurrence of EA following surgery can be anticipated in patients, allowing for the implementation of preventive measures [15].

Age, male sex, smoking, substance abuse history, inhalational anesthesia, urinary catheter use, pain, and the need for analgesic drug use in the PACU were identified as seven risk factors according to a meta-analysis by Wei et al., which included nearly 17,000 patients [13]. Recovery agitation is observed at a higher rate in patients receiving inhalational anesthesia. However, in our patient group, maintenance with intravenous anesthesia was deemed appropriate as it did not disrupt stable hemodynamics and neuromonitoring responses. In patients without neuromuscular monitoring, either method could have been used. For example, in one study, a higher incidence of emergence agitation (EA) was observed in ENT surgeries, with rates of 23.3% in inhalational anesthesia compared to 12.2% in TIVA [10]. To ensure standardization, we included only the TIVA group. Additionally, in patients requiring vasoactive drugs or those with hemodynamic instability, BIS values could have been inaccurately measured. In this study, which included hemodynamically stable patients undergoing elective surgery, emergency cases with a potentially higher risk of emergence agitation were not included. Each of these points could also be the subject of a new study.

Heily et al. [16] assessed EA using the Richmond scale, whereas Abitağaoğlu et al. [17] utilized the Riker scale in their study. These scales are known to be consistent and useful in both contexts. In a study conducted on 104 patients, assessment was performed using the Nursing Instrument for the Communication of Sedation, with the Richmond scale used as a reference [18]. Babar A. Khan et al. demonstrated that these two scales showed consistent similarity in approximately 2500 patients [19]. We evaluated patients using the RASS, with which we are more familiar. RASS is not a continuous measurement, which may lead to missing rapid changes in agitation or sedation. Additionally, factors such as pain, medications, or even conscious attempts to express discomfort can influence measurements. Moreover, assessments can be subjective. These limitations are inherent to the RASS scale, but until a more practical method is developed, it remains the most suitable tool available. As a solution, more frequent assessments may be conducted instead of relying on single scoring instances.

As anesthesiologists, our primary focus is the brain, and EEG serves as one of our most valuable monitoring tools. Numerous benefits, including improved recovery, neuroprotection, and reduced total drug consumption, have been demonstrated with processed EEG monitoring [19]. BIS has important contributions to patient recovery. According to results reported by Sommer et al. from records of approximately 5000 patients, follow-up with processed EEG was shown to reduce the risk of postoperative delirium (POD). Although the pathophysiological mechanisms are not fully understood, the overall benefit in preventing POD has been consistently demonstrated across multiple studies [20].

### 4.1. Relationships Between EA, Recovery, Delirium, and BIS

In a study examining the relationship between processed EEG and POD, Soehle et al. suggested that risk was increased in patients with right-lateralized EEG power and that inhomogeneous EEG changes were also associated with POD. In our study and routine practice, unilateral EEG monitoring was performed [21]. In the same study, POD was more frequent in patients with high burst suppression and longer suppression durations, which was attributed to higher anesthetic consumption. In our study group, however, propofol and remifentanil doses did not differ between groups. Individual patient characteristics may have contributed to suppression susceptibility. Kertai et al. also demonstrated an association between cumulative low EEG time and increased mid-term mortality in patients with comparable anesthetic consumption [22].

These findings further emphasize the importance of EEG-guided anesthesia rather than reliance on standard anesthetic dosing. Since our target BIS range was 40–60, mean perioperative BIS values were similar between patients who developed EA and those who did not. Surgery duration and eye-opening times were also comparable. The Balanced Anaesthesia Study showed no difference in mortality between light (BIS 50) and deep (BIS 35) anesthesia, suggesting comparable outcomes between these depths.

Kertai et al. selected a minimum BIS target of 45 in their delirium-focused study, which included cardiac surgery patients with higher rates of hypothermia [22]. Evered et al., in their comparison of BIS targets of 35 and 50 in a part of Balanced Anaesthesia Study, reported better one-year cognitive outcomes and lower POD risk in patients targeted at BIS 50 [11,23]. Thus, the traditional BIS threshold of 40 may warrant reconsideration in relation to POD risk [9]. BIS suppression was not adequately accounted for in these studies [23]. Without burst suppression data, the predictive value of BIS for delirium and EA may be limited, even when monitored intermittently.

Several studies have suggested that prolonged and severe EEG suppression is associated with unfavorable postoperative outcomes. Yoon et al. demonstrated that low BIS values (<40) combined with hypotension (MAP < 50 mmHg) doubled 180-day mortality [24]. Similarly, low BIS values were associated with increased mortality in patients following cardiac arrest [25]. While many studies have linked low BIS values to hypotension, we found no studies specifically comparing suppression duration with suppression magnitude. Given that our primary outcome was emergence agitation, we focused on the impact of early BIS suppression on EA. Long-term outcomes in patients who develop EA warrant further investigation.

### 4.2. Clinical Outcomes of BIS-Measured EEG Suppression

Dragovic et al. reported that alpha/beta band power of 7.79 and EEG suppression during recovery were associated with lower postoperative delirium incidence [26]. Postoperative delirium was not assessed in our study, as it was beyond our scope. However, unlike Dragovic et al., we found no differences in mean, minimum, or maximum perioperative BIS values, nor in suppression duration or magnitude, between patients who did and did not develop EA. While Dragovic et al. suggested that greater EEG suppression may reduce POD risk, our findings indicate that low suppression alone is insufficient to predict EA risk. This raises the question of whether lower EEG targets should be considered in patients at high risk for EA, and whether maintaining a narrow EEG range (e.g., 35–40) without inducing suppression is feasible—an area for future research.

In both our study and that of Dragovic et al., intraoperative awareness was not evaluated. In the ENGAGES study, none of the 1232 patients reported awareness, and no difference in delirium rates was found between standard and EEG-guided anesthesia groups. In that study, BIS values below 40 were avoided, and inhalational anesthesia was used, with MAC likely supporting anesthesia depth. In our study, anesthesia was maintained with propofol and remifentanil, and BIS values were used alongside hemodynamic parameters to assess depth of anesthesia [27]. Intraoperative unwanted movement occurred in 22% of patients in the BIS group. The absence of an upper BIS limit may have influenced these findings. While ENGAGES concluded that EEG guidance did not reduce delirium but did reduce anesthetic consumption and suppression, other studies, including the Balanced Anaesthesia Study, have challenged this interpretation. Emergence agitation was not evaluated in either study [28,29].

Concerns remain regarding the RASS scoring system, particularly the grouping of “restless” patients (RASS + 1). Alternative scales may allow better differentiation between calm and agitated states. Although our approach aligns with the existing literature, using an additional classification system alongside RASS could have strengthened our analysis. Moreover, RASS’s limited ability to capture instantaneous agitation episodes is an inherent limitation of the scale itself.

BIS values were recorded intermittently rather than continuously, which may be considered a limitation. However, suppression ratios and suppression magnitude were captured in real time and constituted the primary exposure variables in our analysis. Several potential confounders known to influence postoperative emergence agitation such as intraoperative mean arterial pressure, temperature, and ventilation parameters including end-tidal CO_2_ were not incorporated into the primary analyses in a detailed, time-resolved manner.

The use of adjunct medications (e.g., benzodiazepines, ketamine, dexmedetomidine), antiemetics, and neuromuscular blockade reversal agents was not systematically analyzed. In addition, postoperative pain management strategies, opioid or sedative administration, and PACU course were not evaluated, as the scope of the present study was limited to emergence agitation occurring during the immediate recovery period. The potential contribution of PACU-related factors to agitation therefore remains an important area for future investigation and may warrant a separate, dedicated study.

Preoperative neurocognitive status was not formally assessed; consequently, BIS suppression effects were evaluated across the entire cohort irrespective of baseline cognitive function, which represents an additional limitation. Finally, while our analysis primarily focused on ROC-based discrimination, the absence of a multivariable regression model adjusting for prespecified perioperative covariates limits causal inference. Future studies incorporating comprehensive baseline and perioperative management data and multivariable modeling approaches are needed to better delineate the independent contribution of BIS suppression to postoperative emergence agitation.

Total intravenous anesthesia was preferred to ensure stable hypnotic control and to avoid potential interference of volatile anesthetics with neuromonitoring signals and BIS-derived parameters [30]. Remifentanil infusion rates were dynamically adjusted according to surgical stage, anticipated nociceptive intensity, and hemodynamic responses, recognizing the stage-dependent variability in nociceptive load during lumbar spine surgery. Although remifentanil is associated with opioid-induced hyperalgesia, it remains one of the most commonly used intraoperative opioids for spine surgery in our national practice due to its rapid titratability and predictable recovery profile. Restricting the cohort to TIVA-based anesthesia allowed for a more homogeneous evaluation of BIS suppression metrics in this observational study. Future studies may explore whether similar BIS-related outcomes are observed under volatile or combined anesthetic techniques.

With the increasing use of perioperative EEG monitoring, the impact of abnormal EEG patterns on patient outcomes remains an important research area. The long-term effects of non-ideal BIS values, their interaction with fluid status, pain, cerebral perfusion, and their role in patients with neurological disease continue to warrant further investigation using BIS and suppression ratio metrics.

## 5. Conclusions

In this prospective observational study, both the duration and the magnitude of intraoperative EEG suppression measured by the BIS Suppression Ratio demonstrated comparable predictive value for postoperative sedation, while neither parameter was associated with emergence agitation. Conventional BIS values alone were insufficient to differentiate recovery phenotypes, whereas suppression-related metrics provided additional clinically relevant information. These findings suggest that monitoring BIS-derived suppression parameters may enhance perioperative EEG-guided anesthesia by improving anticipation of sedated recovery patterns. Further studies are warranted to clarify optimal suppression thresholds and to determine the broader clinical implications of suppression burden on postoperative and long-term neurological outcomes.

## Figures and Tables

**Table 1:** Comparison of Parameters Across Groups.

Variable	Group 1 (n = 75) Median (IQR)	Group 2 (n = 17) Median (IQR)	Group 3 (n = 49) Median (IQR)	Total (n = 141) Median (IQR)	*p*-Value *
**Age** (years)	50 (20) ^a^	63 (21) ^b^	51 (18) ^a^	50 (21)	0.235
**Sex** (m/f)	34/65% ^a^	41.2%58.8% ^a^	63.3/36.7% ^b^	45.5/54.6%	0.007
**Preoperative BIS**	88(14)	87(5)	88(10.75)	88 (10)	0.903
**Minimum BIS Values**	36 (5)	36 (4)	38 (3.5)	37 (5)	0.154
**Maximum BIS Values**	59 (8)	57 (12)	57 (9.5)	58 (9)	0.364
**SRmax**	28 (12) ^a^	18 (20) ^b^	18 (18.5) ^b^	23 (16)	<0.001
**SRT**	12 (8) ^a^	12 (11) ^a^	10 (9) ^a^	12 (8.5)	0.004
**Op. Duration** (min)	168 (50) ^a^	150 (62.5) ^b^	145 (50) ^b^	155 (47.5)	0.011
**Propofol Dosage** (mg)	108 (22)	98 (27)	103 (25.5)	106 (24)	0.100
**Remifentanil** (µg/kg/min)	0.62 (0.54)	0.55 (0.27)	0.71 (0.45)	0.62 (0.45)	0.092
**Recovery Time** (min)	13 (6) ^a^	10 (6.5) ^b^	10 (9) ^b^	11.92 (8)	0.001

BIS, Bispectral Index; Groups 1–3, recovery with sedation, normal recovery, and emergence agitation, respectively; SRmax, maximum suppression ratio during surgery; SRT, total suppression time. * Statistically significant (*p* < 0.05). Comparisons among the three groups were performed using the Kruskal–Wallis test. When a significant overall difference was identified, pairwise comparisons were conducted using the Mann–Whitney U test with appropriate adjustment for multiple comparisons. ^a,b^: Values sharing the same letter within the same row do not differ significantly.

## Data Availability

The datasets generated and/or analysed during the current study are not publicly available due [requiring written permission from the institution] but are available from the corresponding author on reasonable request.

## Abbreviations

The following abbreviations are used in this manuscript:

BIS	Bispectral Index
EEG	Electroencephalogram
BIS-SR	Bispectral Index Suppression Ratio
SR	Suppression Ratio
SRmax	Maximum Suppression Ratio
SRT	Suppression Ratio Time (Total Suppression Time)
EA	Emergence Agitation
RASS	Richmond Agitation–Sedation Scale
POD	Postoperative Delirium
TIVA	Total Intravenous Anesthesia
